# Millennial Climatic Fluctuations Are Key to the Structure of Last Glacial Ecosystems

**DOI:** 10.1371/journal.pone.0061963

**Published:** 2013-04-16

**Authors:** Brian Huntley, Judy R. M. Allen, Yvonne C. Collingham, Thomas Hickler, Adrian M. Lister, Joy Singarayer, Anthony J. Stuart, Martin T. Sykes, Paul J. Valdes

**Affiliations:** 1 School of Biological and Biomedical Sciences, Durham University, Durham, United Kingdom; 2 Biodiversity and Climate Research Centre (BiK-F), Senckenberg Gesellschaft für Naturforschung, Frankfurt am Main, Germany; 3 Department of Physical Geography, Goethe-University Frankfurt, Frankfurt am Main, Germany; 4 Palaeontology Department, Palaeontology Research Division, Natural History Museum, London, United Kingdom; 5 School of Geographical Sciences, University of Bristol, Bristol, United Kingdom; 6 Department of Physical Geography and Ecosystem Science, Geocentrum 2, Lund University, Lund, Sweden; Odum School of Ecology, University of Georgia, United States of America

## Abstract

Whereas fossil evidence indicates extensive treeless vegetation and diverse grazing megafauna in Europe and northern Asia during the last glacial, experiments combining vegetation models and climate models have to-date simulated widespread persistence of trees. Resolving this conflict is key to understanding both last glacial ecosystems and extinction of most of the mega-herbivores. Using a dynamic vegetation model (DVM) we explored the implications of the differing climatic conditions generated by a general circulation model (GCM) in “normal” and “hosing” experiments. Whilst the former approximate interstadial conditions, the latter, designed to mimic Heinrich Events, approximate stadial conditions. The “hosing” experiments gave simulated European vegetation much closer in composition to that inferred from fossil evidence than did the “normal” experiments. Given the short duration of interstadials, and the rate at which forest cover expanded during the late-glacial and early Holocene, our results demonstrate the importance of millennial variability in determining the character of last glacial ecosystems.

## Introduction

Fossil remains of mega-herbivores, including the iconic Woolly Mammoth (*Mammuthus primigenius*) and Woolly Rhinoceros (*Coelodonta antiquitatis*), show they were widespread and abundant in ice-free areas of the northern continents during the last glacial [1], [2]. Such large-bodied grazers require productive ecosystems dominated by herbaceous plants. Although macrofossils indicate trees were locally present [3], pollen data indicate the glacial vegetation throughout most of Europe [4], northern Asia [5], Beringia [6] and western North America [7] was dominated by non-woody plants, and thus potentially able to support mega-herbivores. Fossil evidence, however, provides limited, if any, quantitative evidence of vegetation productivity, and hence of its capacity to sustain a grazer community.

Vegetation models provide a means to estimate structure, composition and productivity of glacial vegetation. To apply such models, however, glacial climatic conditions are required as input, in turn requiring output from GCM palaeoclimate experiments. GCM experiments made with prescribed sea surface temperatures and sea-ice extent [8] gave results that disagreed with pollen and other palaeoclimatic evidence, especially in western Europe [4], [9]. When these palaeoclimate experiments were used to drive an equilibrium vegetation model, the glacial vegetation of Europe was simulated to be largely forest, a result that conflicts strongly with pollen evidence [4]. More recent experiments with fully-coupled atmosphere–ocean GCMs [10], [11] give results that conflict much less with geological evidence of last glacial climates. In parallel, dynamic vegetation models have been developed [12], [13] that simulate the processes of vegetation development and its responses not only to climatic changes but also to changes in atmospheric CO_2_ concentration and in seasonal solar radiation intensity. One such model, LPJ-GUESS [14], has been used to simulate last glacial vegetation of northern Eurasia [15], simulations being driven by outputs from palaeoclimate experiments made using the HadCM3 GCM [10]. Although these simulations showed generally limited forest extent before and during the LGM, productivity of woody plants in western Eurasia at the LGM was higher than expected and inconsistent with very sparse representation of trees in the pollen record. Rapid extension of simulated forest cover across Eurasia after the LGM broadly coincided with rapid geographical range reductions of previously widespread mega-herbivores, and was likely to have been a major contributor to the extinction of many [2], but relatively low simulated productivity of herbaceous plants at the LGM, especially in the west, conflicted with evidence of widespread presence and abundance of mega-herbivores.

Ice-core records show glacial climates were characterised by millennial-scale alternations between warmer interstadials and colder stadials [16]. The most extreme alternations were associated with Heinrich Events in the North Atlantic [17], during which Atlantic meridional overturning circulation apparently collapsed [18]. Comparing results of the HadCM3 experiments with temperatures reconstructed for central Greenland shows that experiments using a combination of ‘slow’ forcings (orbital configuration; atmospheric composition; ice sheet extent and topography; sea-level; and land–sea mask), referred to hereafter as ‘normal’ experiments, generally match and track temperatures reconstructed for the warmest parts of interstadials [10]. In contrast, a series of ‘hosing’ experiments [10] designed to mimic Heinrich Events H1 to H5 give temperatures close to and tracking those reconstructed for stadials. LPJ-GUESS simulations driven by the normal experiments thus indicate potential vegetation of the warmest parts of interstadials. They hence give a biased view of last glacial vegetation, because peak interstadial conditions account for a minority of the time.

To investigate potential stadial vegetation, and how this differs from that of peak interstadials, we performed two series of LPJ-GUESS simulations, the first driven by the five hosing experiments and the second driven by the normal experiments for the time slices corresponding to the Heinrich Events (H1–17 ka BP; H2–24 ka BP; H3–32 ka BP; H4–38 ka BP; H5–46 ka BP). A further pair of simulations, for 6 ka BP and 120 ka BP, was performed to explore how vegetation patterns during the last interglacial may have differed from those during the Holocene; in particular, whether they were more favourable for survival of mega-herbivores, at least in East Siberia [19], during the last interglacial.

## Materials and Methods

Methods for the LPJ-GUESS simulations were as in Reference [15], with the following two exceptions. Firstly, simulations were made for the entire northern hemisphere land area north of 35°N. Secondly, simulations were extended to include continental shelf areas exposed by glacial sea-level depression. To achieve the latter, 1961–90 climatic conditions were estimated for shelf areas by interpolation and/or extrapolation of the CRU CL 1.0 data [20], as described in Reference [21], and palaeoclimatic conditions inferred using the same anomaly-based approach used in the earlier work [15], thus minimising impacts of GCM biases. Results were summarised by summing simulated annual net primary productivity (ANPP) values for each grid cell and experiment for three groups of plant functional types (PFTs):trees–woody PFTs of tree stature, taken to be ≥3 m; shrubs–woody PFTs forming only shrubs (≤3 m tall) or dwarf shrubs; and herbs–non-woody PFTs.

## Results

**Figure 1:**
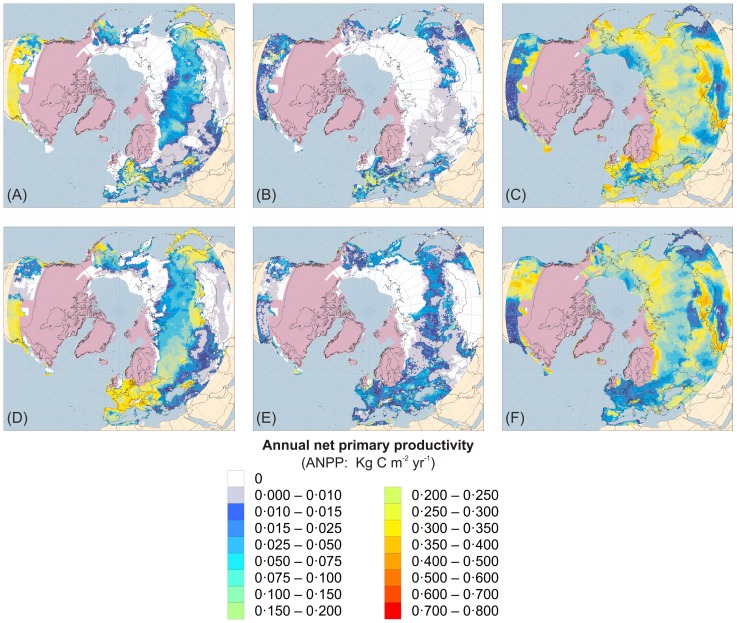
Tree, shrub and herb plant functional type ANPP:Heinrich Event 1 hosing experiment compared to 17 ka BP normal experiment. Annual net primary productivity (ANPP) for the aggregated tree (A and D), shrub (B and E) and herb (C and F) plant functional types (PFTs) simulated for the palaeoclimates generated by the Heinrich Event 1 hosing experiment (A–C) and the equivalent 17 ka BP normal experiment (D–F). Lilac shaded areas indicate the extent of the modelled last glacial ice sheets for 17 ka BP [38]; land area is shown for sea-level lowered by 107 m [39]. ANPP is indicated by shading of land areas:white areas have zero ANPP for that PFT; pale grey areas have non-zero but very low ANPP; shades from deep blue through cyan and pale cyan to yellow and orange indicate progressively higher ANPP as shown by the quantitative legend.

**Figure 2:**
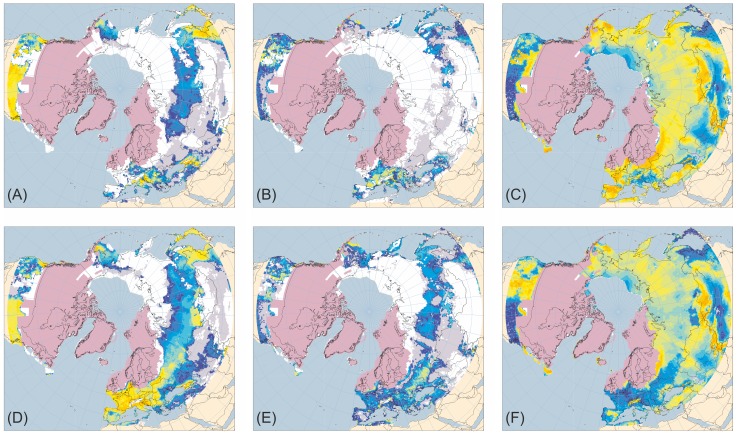
Tree, shrub and herb plant functional type ANPP: Heinrich Event 2 hosing experiment compared to 24 ka BP normal experiment. ANPP for the aggregated tree (A and D), shrub (B and E) and herb (C and F) plant functional types (PFTs) simulated for the palaeoclimates generated by the Heinrich Event 2 hosing experiment (A–C) and the equivalent 24 ka BP normal experiment (D–F). Lilac shaded areas indicate the extent of the modelled last glacial maximum ice sheet [38]; land area is shown for sea-level lowered by 112 m [40]. ANPP is indicated by shading of land areas: white areas have zero ANPP for that PFT; pale grey areas have non-zero but very low ANPP; shades from deep blue through cyan and pale cyan to yellow and orange indicate progressively higher ANPP (for quantitative legend see Fig. 1).

**Figure 3:**
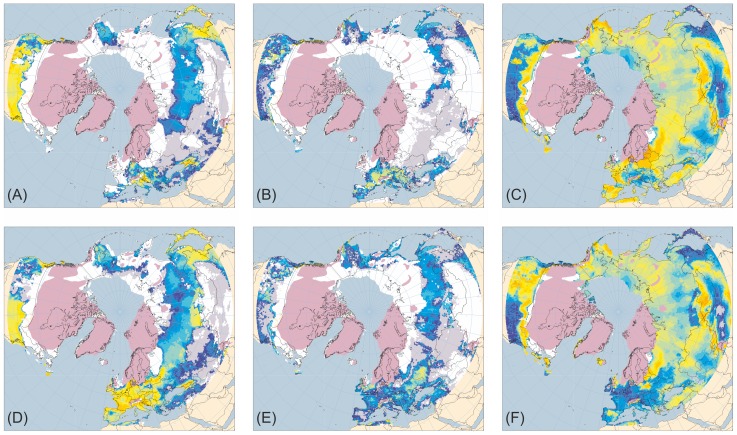
Tree, shrub and herb plant functional type ANPP: Heinrich Event 3 hosing experiment compared to 32 ka BP normal experiment. ANPP for the aggregated tree (A and D), shrub (B and E) and herb (C and F) plant functional types (PFTs) simulated for the palaeoclimates generated by the Heinrich Event 3 hosing experiment (A–C) and the equivalent 32 ka BP normal experiment (D–F). Lilac shaded areas indicate the extent of the ice sheets mapped for the mid-Weichselian [41]; land area is shown for sea-level lowered by 80 m [40]. ANPP is indicated by shading of land areas: white areas have zero ANPP for that PFT; pale grey areas have non-zero but very low ANPP; shades from deep blue through cyan and pale cyan to yellow and orange indicate progressively higher ANPP (for quantitative legend see Fig. 1).

**Figure 4:**
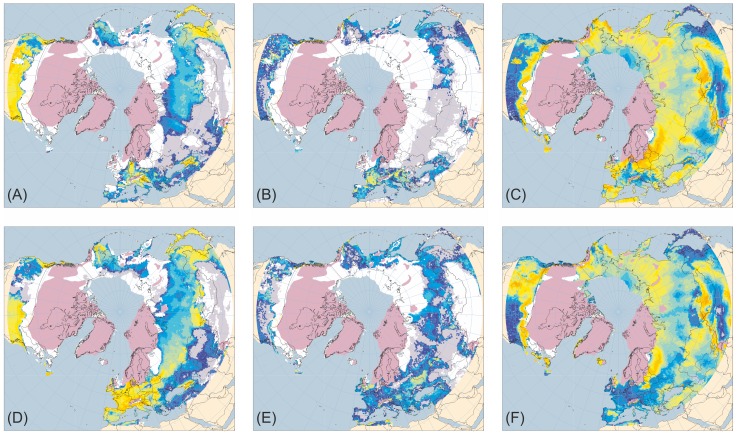
Tree, shrub and herb plant functional type ANPP: Heinrich Event 4 hosing experiment compared to 38 ka BP normal experiment. ANPP for the aggregated tree (A and D), shrub (B and E) and herb (C and F) plant functional types (PFTs) simulated for the palaeoclimates generated by the Heinrich Event 4 hosing experiment (A–C) and the equivalent 38 ka BP normal experiment (D–F). Lilac shaded areas indicate the extent of the ice sheets mapped for the mid-Weichselian [41]; land area is shown for sea-level lowered by 80 m [40]. ANPP is indicated by shading of land areas: white areas have zero ANPP for that PFT; pale grey areas have non-zero but very low ANPP; shades from deep blue through cyan and pale cyan to yellow and orange indicate progressively higher ANPP (for quantitative legend see Fig. 1).

**Figure 5:**
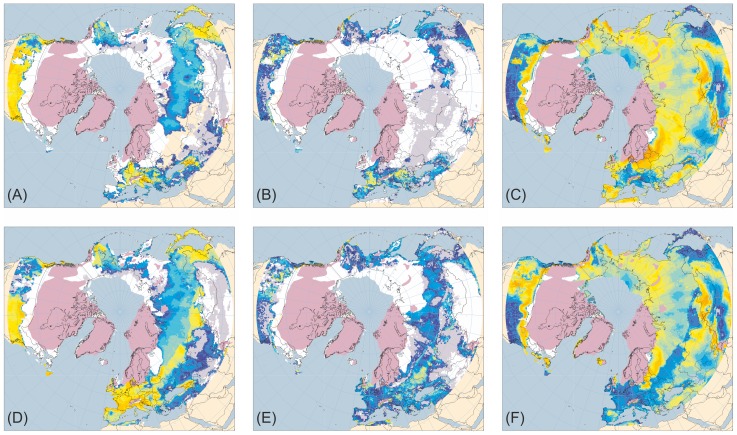
Tree, shrub and herb plant functional type ANPP: Heinrich Event 5 hosing experiment compared to 46 ka BP normal experiment. ANPP for the aggregated tree (A and D), shrub (B and E) and herb (C and F) plant functional types (PFTs) simulated for the palaeoclimates generated by the Heinrich Event 5 hosing experiment (A–C) and the equivalent 46 ka BP normal experiment (D–F). Lilac shaded areas indicate the extent of the ice sheets mapped for the mid-Weichselian [41]; land area is shown for sea-level lowered by 80 m [40]. ANPP is indicated by shading of land areas: white areas have zero ANPP for that PFT; pale grey areas have non-zero but very low ANPP; shades from deep blue through cyan and pale cyan to yellow and orange indicate progressively higher ANPP (for quantitative legend see Fig. 1).

**Figure 6:**
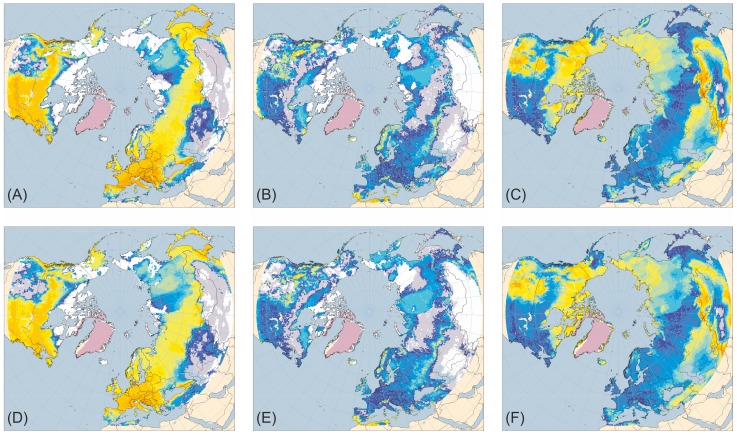
Tree, shrub and herb plant functional type ANPP: 120 ka BP (Last interglacial) compared to 6 ka BP (Holocene ‘optimum’). ANPP for the aggregated tree (A and D), shrub (B and E) and herb (C and F) plant functional types (PFTs) simulated for the palaeoclimates generated by the 120 ka BP (last interglacial) experiment (A–C) and the 6 ka BP (Holocene ‘optimum’) experiment (D–F). Lilac shaded areas on the maps for 6 ka BP indicate the extent of the modelled ice sheets for that time [38] whilst those on the maps for 120 ka BP show the extent of ice sheets mapped for the Holocene by Ehlers and Gibbard [41]; land area is shown for sea-levels of −8 m [39] and +2 m [40] for 6 ka BP and 120 ka BP, respectively. ANPP is indicated by shading of land areas: white areas have zero ANPP for that PFT; pale grey areas have non-zero but very low ANPP; shades from deep blue through cyan and pale cyan to yellow and orange indicate progressively higher ANPP (for quantitative legend see Fig. 1).

Figures 1–5 present the results for H1/17 ka BP, H2/24 ka BP, H3/32 ka BP, H4/38 ka BP and H5/46 ka BP respectively. In each case, across large areas of northern Eurasia, but especially Europe, hosing experiment climates give generally lower ANPP for trees and shrubs and higher ANPP for herbs than equivalent normal experiments. This is in much better accord with pollen evidence than is vegetation simulated for normal experiments. In contrast, hosing experiments give higher simulated tree ANPP in western North America south of the Laurentide Ice Sheet than do normal experiments. This too accords better with fossil evidence, areas of highest simulated ANPP matching areas of highest reconstructed tree density [7]. Figure 6 shows the results for 6 and 120 ka BP and indicates that East Siberia had higher ANPP of herbaceous PFTs and lower ANPP of woody PFTs during the last interglacial than during the Holocene.

## Discussion

Given greater prevalence of stadial conditions, and relatively limited duration of the warmest parts of most interstadials, the contrast in simulated vegetation between hosing and normal experiments provides the key to reconciling modelled vegetation with the fossil record, and also to understanding the nature of glacial ecosystems that supported mega-herbivore communities. Under stadial conditions, ANPP of herbaceous plants was increased, whereas that of trees was much reduced, especially in Europe, and was less than that of herbs. It is likely that trees were only sparsely present in most areas, either as scattered individuals or localised stands, in a landscape dominated by productive herbaceous communities. Furthermore, tree ANPP was non-zero north of the Alps principally in central Europe, corresponding to the location of macrofossil occurrences of trees, and only Boreal tree PFTs contributed, again matching macrofossil data [3], [22]. On each occasion when interstadial conditions developed, temperate trees, limited to southern Europe during the last glacial [23], would begin to expand their ranges into newly suitable areas from which they were excluded during stadials. Populations of Boreal trees able to persist north of the Alps under stadial conditions would expand across the landscape from the favourable sites in which they had survived. Late-glacial and Holocene pollen data provide a basis for estimating rates at which such expanding tree populations in turn lead to the expansion of forest or woodland cover [24], [25]. Although forests or woodlands formed by a minority of taxa were able to expand at rates of between 1 and 2 km yr^−1^, long-term average expansion rates of forests formed by most tree taxa were between 200 and 500 m yr^−1^. Most trees hence require two to five millennia to advance the margin of the area where they form extensive forest cover by 1000 km; even the most rapid require 500 yr to achieve this distance.

Most last-glacial interstadials, however, were of short duration, especially during marine oxygen isotope Stage 2. Fifteen interstadials with discrete onset and termination have durations between 100 and 2600 yr (mode 300 yr; mean 1000 yr) [16]. At NGRIP, last glacial (14·7–76·5 b2k) δ^18^O values are in the upper 25% of the range for that interval only 9% of the time, whereas they are in the lower half of the range 61% of the time (data file “2010-11-19 GICC05modelext for NGRIP.xls” downloaded 11/1/12 from http://www.iceandclimate.nbi.ku.dk/data/). Many areas simulated as more or less treeless under stadial conditions extend >1000 km from areas with higher ANPP of trees under stadial conditions. Most trees are thus unlikely to have expanded their ranges and populations sufficiently to develop the extents of forest or woodland cover potentially possible under interstadial climatic conditions, except perhaps during the longest interstadials (GI 12: 46·8–44·2 ka BP; GI 19: 72·3–70·3 ka BP; and GI 20: 76·4–74·0 ka BP). Glacial vegetation was thus, as hypothesised by Lister and Sher [26], maintained in a generally treeless but productive state across most of the northern continents as a result of what they referred to as “the constant stirring” of millennial climatic fluctuations.

These modelling results and the inferences we make from them are fully consistent with the palaeovegetation evidence from Europe for the last glacial stage. Allen and Huntley [27] reviewed the then available evidence and showed that at sites in southern Europe, south of the main mountain chains, tree pollen abundance increased to levels indicative of open woodland or wooded steppe during last glacial interstadials, whereas grasses and steppic herbs predominated during stadial intervals. During the longer and warmer interstadials between *ca*. 60,000 cal yr BP and *ca*. 30,000 cal yr BP, temperate forest trees were important components of the woody vegetation, whereas the shorter and colder interstadials that predominated between *ca*. 75,000 cal yr BP and *ca*. 60,000 cal yr BP, as well as between *ca*. 30,000 cal yr BP and *ca*. 14,000 cal yr BP, generally were characterised by the predominance of *Pinus* and *Juniperus* amongst the woody taxa. North of the main mountain chains, most interstadials are expressed more weakly in the palaeovegetation record. Increased amounts of tree pollen at sites in central Europe indicate development of open woodland during some of the longer and warmer intervals, although the tree taxa concerned are restricted to Boreal taxa, notably *Betula*, *Picea*, *Pinus* and *Juniperus*. In northern Europe the palaeovegetation record documents only a few of the interstadial events; they are characterised there by a predominance of herbaceous and dwarf-shrub taxa, with pollen of woody taxa infrequent, principally being very low abundances of Boreal taxa, notably *Betula*, *Pinus* and *Juniperus*, the first and last of which are likely to represent principally the shrubby taxa *B. nana* and *J. communis*. Although a few additional records, for example Reference [28], have been published since the review by Allen and Huntley [27] was undertaken, these records have not altered in any substantive way the overall picture. Forest did not extend across Europe north of the main mountain chains at any time during Marine Oxygen Isotope (MOI) stages 2–4 [29], although during interstadials woodland or wooded steppe with temperate tree taxa extended across areas of southern Europe, especially during MOI stage 3. Even during the latter warmer and/or longer interstadials, however, only Boreal tree populations expanded in central Europe, forming open or patchy woodlands rather than extensive forest cover, whilst in northern Europe herbaceous and dwarf-shrub vegetation prevailed during these intervals.

In contrast to the last glacial, Holocene climatic fluctuations have been generally of much smaller magnitude than those during the glacial. Conditions favourable for forest development across most of northern Eurasia have prevailed throughout the Holocene. The climatic fluctuations that have occurred generally have resulted in only relatively small shifts (≤100 km) in the latitudinal treeline, altering the overall extent of forest cover by *ca*. 5% at most. Thus, not only have conditions favoured extensive forest development in areas where herbaceous taxa dominated glacial ecosystems [15], but forests have persisted more than ten millennia in many areas. The relative rapidity with which forests extended, compared to the rate of marginal retreat of the Laurentide and Fennoscandian Ice Sheets, resulted in a critical late-glacial to early Holocene reduction in extent of cold, productive herbaceous ecosystems upon which the mega-herbivores depended. For some, e.g. Woolly Rhinoceros–*Coelodonta antiquitatis*, extinction followed initial forest expansion after the LGM [2]. Others, e.g. Woolly Mammoth–*Mammuthus primigenius*, also contracted their range greatly around this time but persisted to the mid-Holocene, in this case in restricted areas of suitable habitat on islands north of Siberia [30] and in the Bering Sea [31], but eventually went extinct. Yet others of the glacial mega-herbivore assemblage are extant, e.g. Musk Oxen–*Ovibos moschatus*, but show genetic evidence of a severe bottleneck between the LGM and late Holocene [32], only occupying their principal modern range in Greenland after the mid-Holocene [33].

In contrast to the Holocene, last interglacial palaeovegetation evidence from East Siberia indicates continuous forest cover was less extensive in that region [5], [34] during that interval. Consistent with this, simulated vegetation in East Siberia for 120 ka BP shows generally higher ANPP of herbs and lower ANPP of woody PFTs, especially trees, than at 6 ka BP (Fig. 6) Notwithstanding higher simulated tree ANPP in many Boreal and temperate forest areas at 120 ka BP than at 6 ka BP, the Arctic treeline generally is simulated at a lower latitude at 120 ka BP than at 6 ka BP. Local exceptions include an area of higher tree ANPP at 120 ka BP close to the East Siberian north coast, corresponding to evidence of occurrence of trees in that area [34]. In contrast to the Holocene, last interglacial ecosystems in East Siberia were thus probably able to support mega-herbivore communities. Such contrasts between interglacials are to be expected, given the differences in orbital forcing and hence in climatic conditions. Furthermore, the contrast in vegetation characteristics of East Siberia between the last interglacial and the Holocene provides support for the argument that the particular nature of the environmental changes after the last glacial maximum and during the early Holocene was the primary factor in causing the extinctions of many larger vertebrates of Eurasia during that period, rather than the coincidental increase in the geographical range, abundance and/or technological status of anatomically modern humans [35].

Climate is once again changing rapidly [36], with implications for biodiversity that are of global concern. Our results provide the basis for either optimism or pessimism, depending upon predictions of how climate is likely to evolve over coming centuries. If the recent and continuing perturbation of atmospheric composition, and associated global mean temperature increase, is an excursion, with a return to pre-industrial levels of greenhouse gases in the atmosphere within the next century or so, then, as during the last glacial, we can expect the limited response rates of many ecosystems, and forest systems especially, to provide a buffer against more extreme impacts upon biodiversity. If, on the other hand, as seems very likely, atmospheric composition remains in a perturbed state, and global mean temperature as a result increases to a value more than *ca*. 2°C warmer than its pre-industrial value, we can expect major ecosystem disruption and associated extinctions [37], as occurred with the shift to persistently warmer conditions at the onset of the Holocene.
